# Comparative Network Pharmacology Analysis of Classical TCM Prescriptions for Chronic Liver Disease

**DOI:** 10.3389/fphar.2019.01353

**Published:** 2019-11-22

**Authors:** Zikun Chen, Xiaoning Wang, Yuanyuan Li, Yahang Wang, Kailin Tang, Dingfeng Wu, Wenyan Zhao, Yueming Ma, Ping Liu, Zhiwei Cao

**Affiliations:** ^1^Department of Gastroenterology, Shanghai Tenth People's Hospital, School of Life Sciences and Technology, Tongji University, Shanghai, China; ^2^Institute of Interdisciplinary Integrative Medicine Research, Shanghai University of Traditional Chinese Medicine, Shanghai, China; ^3^Department of Pharmacology, Shanghai University of Traditional Chinese Medicine, Shanghai, China; ^4^Key Laboratory of Liver and Kidney Diseases of Ministry of Education of China, Institute of Liver Diseases, Shuguang Hospital, Shanghai University of Traditional Chinese Medicine, Shanghai, China

**Keywords:** network comparative analysis, TCM, chronic liver disease, Yinchenhao Decoction, Huangqi Decoction, Yiguanjian

## Abstract

Chronic liver disease (CLD) has become a major global health problem while herb prescriptions are clinically observed with significant efficacy. Three classical Traditional Chinese Medicine (TCM) formulae, Yinchenhao Decoction (YCHT), Huangqi Decoction (HQT), and Yiguanjian (YGJ) have been widely applied in China to treat CLD, but no systematic study has yet been published to investigate their common and different mechanism of action (MOA). Partial limitation may own to deficiency of effective bioinformatics methods. Here, a computational framework of comparative network pharmacology is firstly proposed and then applied to herbal recipes for CLD disease. The analysis showed that, the three formulae modulate CLD mainly through functional modules of immune response, inflammation, energy metabolism, oxidative stress, and others. On top of that, each formula can target additional unique modules. Typically, YGJ ingredients can uniquely target the ATP synthesis and neurotransmitter release cycle. Interestingly, different formulae may regulate the same functional module in different modes. For instance, YCHT and YGJ can activate oxidative stress-related genes of SOD family while HQT are found to inhibit SOD1 gene. Overall, our framework of comparative network pharmacology proposed in our work may not only explain the MOA of different formulae treating CLD, but also provide hints to further investigate the biological basis of CLD subtypes.

## Introduction

With increasing alcohol consumption and viral infection, chronic liver disease (CLD) has become a worldwide health concern. Chronic liver disease in the clinical context is a disease process of the liver that involves a process of progressive destruction and regeneration of the liver parenchyma leading to fibrosis and cirrhosis. “Chronic liver disease” refers to disease of the liver that lasts over a period of six months. It consists of a wide range of liver pathologies covering inflammation (chronic hepatitis), liver cirrhosis, and hepatocellular carcinoma. According to causes, chronic liver diseases can be classified as viral disease, toxic and drug-related disease, metabolic disease, autoimmune-response disease, and so on. While the nucleotide analogues (NUCs) and pegylated-interferon (Peg-IFN) therapies are effective, patients may not tolerate their adverse effects in lifelong treatment. In the meantime, most of CLD patients seek medical help from herbal medicine for its significant efficacy and low side effects. Recently, several meta-studies of randomized, controlled, clinical trials (RCTs) suggested that herbal remedies have a similar and even better effects compared to western drug therapy in dealing with CLD (Peng et al., 2016; Chen et al., 2019).

According to Guidelines for the diagnosis and treatment of liver fibrosis in integrative medicine practice of China, three classical formulae, Yinchenhao Decoction (YCHT), Huangqi Decoction (HQT), and Yiguanjian (YGJ) have been recommended to treat different subtypes of CLD according to “Zheng” differentiation, which classifies the pathological state of a patient at system level leading to individualized treatments. Syndrome Differentiation and Treatment (SDAT) is the basis of clinical application of traditional Chinese Medicine prescription. A chronic liver disease can be treated with the different efficacy TCM prescription based on its different TCM syndrome. Formula YCHT is suggested to treat “damp-heat” type of patients featured by jaundice, inflammation, and abnormal fatty acids metabolism (Yan et al., 2017), and the therapeutic characteristic of Yinchenhao decoction is to clear heat and drain dampness. Clinically, it is applied mainly to treat patients who have acute jaundice hepatitis, hepatitis B, cholelithiasis, cholecystitis, leptospirosis, typhoid fever, pneumonia, hemolytic jaundice caused by favism, and so on (Yan et al., 2017), while formula HQT, with the proportion of Huangqi [*Astragalus mongholicus* Bge.] and Zhigancao [*Glycyrrhiza uralensis* Fisch.] at 6:1, is recommended for those “Qi-deficiency” patients with weak body, black and yellowish complexion, palpitations and vexations in the chest, dry lips and mouths, pale complexion and lack of appetite. The therapeutic characteristic of HQT is tonifying qi. HQT is applied clinically to treat CLD patients who have liver fibrosis or cirrhosis, heart failure, constipation, type 2 diabetes, and diabetic peripheral neuropathy with “Qi-blood deficiency” (Wang et al., 2015). Additionally, HQT alleviated DMN-induced liver fibrosis (Song et al., 2016). As the therapeutic characteristic of YGJ is nourishing liver and kidney, it is applied clinically to treat those who have chronic liver diseases, chronic gastritis, gastric and duodenal ulcer, intercostal neuralgia, and neurosis with “Yin-deficiency” (Wang et al., 2015).

Studies have investigated the liver-protective activity of above formula. For instance, being composed of Yinchen [*Artemisia capillaris* Thunb.], Zhizi [*Gardenia jasminoides* J. Ellis], and Dahuang [*Rheum palmatum* L.], YCHT demonstrated variable ability of liver protection in treating cholestasis, liver fibrosis, hepatitis, biliary cirrhosis, and cholesteric liver diseases (Yan et al., 2017). The liver protection of YCHT was also confirmed by histopathology and biochemical experiments (Sun et al., 2019), and YCHD treatment could reverse the damage by DMN-induced in liver function (Cai et al., 2018). According to literature validation, pharmacological research revealed that YCHT showed function of anti-oxidative stress, inhibiting apoptosis. Also, it was validated to regulate cellular inflammation and lipid metabolism (Wang et al., 2015). Kupffer cells (KC) activation of inflammatory response and abnormal fatty acid metabolism were found to be the main pathological basis in the early inflammation for YCHT Syndrome (Xu-Dong et al., 2014).

Through literature searching, HQT decoction was reported to downregulate the expressions of PDGF genes, collagen genes (COL1A1, COL1A2, COL5A2), and THBS1, inhibiting TGF-beta and PDGF signaling pathways, followed by verification *via* qRT-PCR (Zhang et al., 2015). Also, it was noticed to promote Kupffer cell activation (Du et al., 2012; Liu et al., 2012; Zhang et al., 2015; Song et al., 2016), inhibit Notch signaling pathway (Wang et al., 2015), and alleviate oxidative stress and lipid peroxidation injury. In an animal experiment, HQT was found to significantly reduce alpha-naphthylisothiocyanate-induced cholestasis in mice (Wu et al., 2017). At herb level, herb Huangqi in HQT were reported with anti-oxidative and immune regulating effects (Shahzad et al., 2016; Li et al., 2019).

YGJ decoction has 6 herbs: Beishashen [*Glehnia littoralis* F. Schmidt ex Miq.], Maidong [*Ophiopogon japonicus* (Thunb.) Ker Gawl.], Danggui [*Angelica sinensis* (Oliv.) Diels], Shengdihuang [*Rehmannia glutinosa* (Gaertn.) DC.], Gouqizi [*Lycium barbarum* L.], and Chuanlianzi [*Melia azedarach* L.]. Research indicates the hepatoprotective, anti-fibrogenic, anti-angiogenesis effects of YGJ in hepatic injury mice models (Tian et al., 2016). In rat model of liver fibrosis, YGJ was found to alleviate the hepatic collagen hyperplasia and deteriorated hepatic function (Tao et al., 2009). Also, YGJ can reduce the liver oxidative stress and lipid peroxidation injury while inhibiting angiogenesis and induce cell differentiation (Zhou et al., 2015). In a rat model of liver cirrhosis, YGJ was reported with repairing function of liver cirrhosis, and the key mechanism was suggested as relating to the regulation of macrophage activation state (Xu et al., 2018).

Above studies showed the biological activity of formulae, but it would be interesting to know the detailed MOA difference or similarities between the three formulae under the same background of CLD disease. As TCM formulae are believed to produce efficacy in a holistic way, the difficulty in detecting the MOA similarities and differences lie in the inherent complexity of multi-ingredients and multi-targets for TCM formulae.

With the development of systems biology and accumulated herbal targets information, the emerging method of network pharmacology makes it possible for MOA analysis of herbal ingredients. Coupled with transcriptomics validation, a latest article provided an excellent model adopting network pharmacology to predict the active components and regulation mechanisms of YCHT against liver fibrosis (Cai et al., 2019). Until now, network pharmacology has been applied to study individual ingredients, herbs, or formulae. One reason causing the difficulty is the lack of effective bioinformatics methods. Here, we developed a computational framework aiming to compare the MOA between formulae for same disease, taking the YCHT, HQT, and YGJ as an example.

The results indicated that although different formulae have different molecular targeting profile, they are commonly involved in a group of functional modules, indicating the CLD disease background. In addition to that, each formula was detected with unique function modules in MOA analysis. More interestingly, even on the same functional module, different regulating modes were found for different formulae agreeing with observed “Zheng” classification of CLD subtyping.

## Materials and Methods

### Dataset

#### Formulae, Herbs, Ingredients, and Targets

For each of the three formulae, herb components, ingredients and targets of each ingredients were collected from HIT (Ye et al., 2011), NPASS (Zeng et al., 2018b), TCMDB (Chen, 2011) and TCM-ID (Huang et al., 2018) databases respectively.

### Method

#### Disease Network, Disease Modules and Function Annotation

Targets of herbal active ingredients were obtained from HIT (Ye et al., 2011) and NPASS (Zeng et al., 2018b) database for target network construction. Potential protein-protein interaction (PPI) network of disease is generated *via* Reactome database (Croft et al., 2014) based on combined unique targets from 3 formulae *via* step size 1 (one bridging node). Then, the disease network was modularized through ReactomeFIViz (Wu and Haw, 2017). Genecards database (Safran et al., 2010) was used for function annotation of each module because of its high level of function annotation. Disease network and modularization were displayed *via* Cytoscape (Shannon et al., 2003). KEGG (Kanehisa, 2002) and GO Biological Process (Ashburner et al., 2000; Mi et al., 2017; The Gene Ontology, 2017) databases were used for biological function annotation for targets of each formula.

#### Statistic Enrichment of Functional Modules

For the given number of formula targets, we tested whether it is significantly enriched in a specific module A, comparing to a randomly picked module with equal size of A, from nodes of disease network background. To make the variation comparable in statistics, we repeated the picking for 5000 times. And each time, half the number of given targets were randomly picked. T-test statistics was made between the hitting of tested targets on specific module and the random module equally sized. According to above, different number of targets for each formula were enriched respectively into the functional modules of disease.

#### Effect of Kaempferol on the Absorption of Astragalosides

To investigate the effect of kaempferol on the absorption of astragalosides, experiments of verted rat sac was performed as previously described (Yan et al., 2015). Briefly, the everted intestinal sacs were prepared by rapidly removing the small intestine from starved rats euthanized under carbon dioxide (CO_2_) anesthesia. Then, four intestinal segments (the duodenum, upper jejunum, bottom jejunum, and ileum) were excised, flushed several times with saline solution at room temperature, and then placed immediately into oxygenated buffer solution at 37°C. Intestine segments from four rats were divided into four groups treated with astragalosides without or with kaempferol at three different concentrations (1, 3, 10M) using a 4 × 4 Latin square design (Gressley et al., 2016).

**Table:** 

4 × 4-latin square design.				
Rat	Duodenum	Upper jejunum	Bottom jejunum	Ileum
1	A	B	C	D
2	B	C	D	A
3	C	D	A	B
4	D	A	B	C


A: Astragalosides + 1µM kaempferol; B: Astragalosides + 3µM kaempferol; C: Astragalosides + 10µM kaempferol; D: Astragalosides.

The concentration of Astragaloside solutions was set at 8 mg/mL based on the gastrointestinal concentration after oral administration of Huangqi [*Astragalus mongholicus* Bge.] decoction to rats, and the concentration of the additional kaempferol was 1, 3, and 10 µM. Then, aliquots of the sac fluid sample (200 µL) were removed, and the same volume of buffer solution was added at 0, 0.5, 1, and 2 h. The area of each sac was calculated closely. Furthermore, each sac was weighed before and after fluid collection to calculate accurately the volume inside the sac. The samples were analyzed using UPLC-LTQ-Orbitrap in both positive and negative ionization mode as previously described. The proposed method could be used to determine the 10 compounds in astragalosides simultaneously with high precision, sensitivity, and accuracy (Zeng et al., 2018a). A multivariate analysis of variance (ANOVA) was performed using the SPSS ver. 13.0 software while p-values < 0.05 were considered statistically significant.

## Results

### Computational Framework of Comparative Network Pharmacology

**Figure 1:**
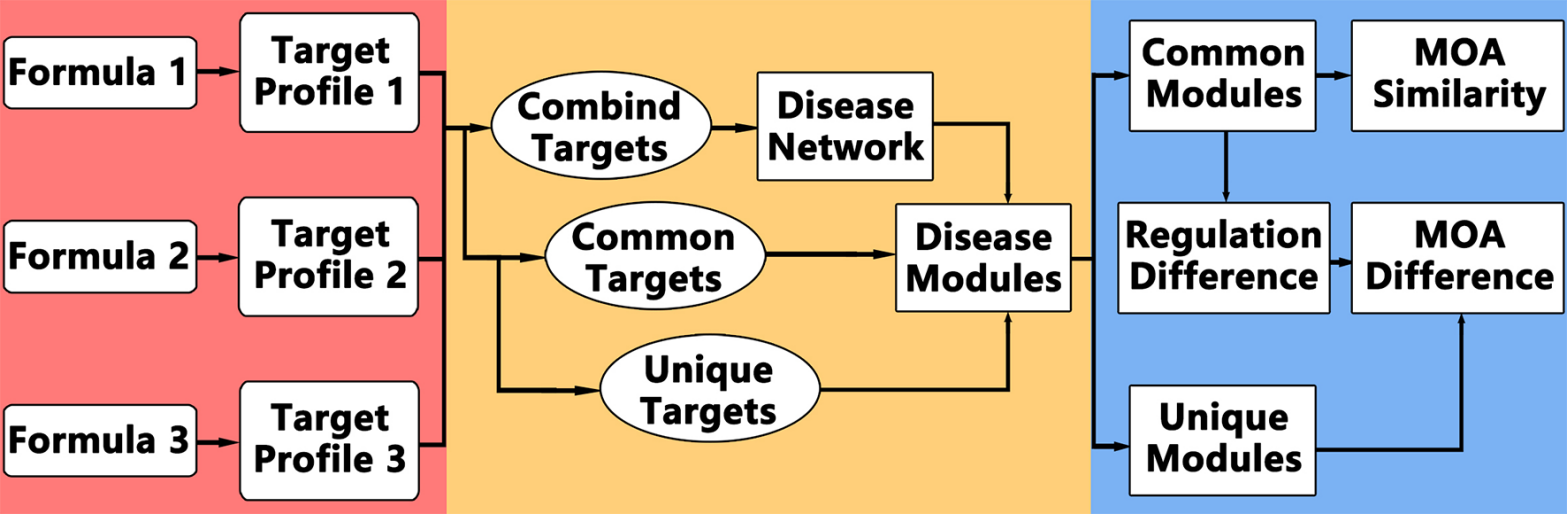
Computational Framework of comparative network pharmacology.

The idea of comparative network pharmacology is to construct a background disease network covering all the targets of different formulae, and then analyze the common and different modules or regulations. The framework of comparative network pharmacology is proposed as below (**Figure 1**): 1. Disease network construction and modularization of combined targets; 2. Comparative analysis of formulae on disease module network; 3. Detailed regulation comparison between formulae.

Accordingly, the computational framework works like this: 1. Retrieve targets of each formula for same disease; 2. Construct the disease network based on combined targets of all formulae; 3. Partition and annotate the disease network into function modules; 4. Calculate the enriched modules for common targets among all formulae and unique targets for each formula; 5. Analyze the MOA similarities and differences.

### Disease Network and Function Modules

The known ingredients with experimental target evidence from literatures were retrieved from databases and summarized into **Supplementary Table 1** for each herb and formula. Though different formulae are composed of different number of herbs, the overall known targets for each formula is roughly similar ranging from 120 to 140 genes. A total of 293 non-redundant targets were combined for all 3 formulae.

Targets of each formula was firstly enriched into KEGG and GO database for function annotation. The results show that all the three formulae are primarily targeting pathway of Hepatitis B, suggesting their common therapeutic effects in liver disease, but the enriched pathway lists are heavily overlapping (**Supplementary Table 2**). Similarly, mapping to GO databases also shows similar pathways profiles and GO terms. This suggests that the commonly used bioinformatics analysis of KEGG and GO annotation is good at inferring the MOA similarities, rather than the MOA differences for different formulae.

After running the pipeline of comparative network pharmacology, we proposed the biggest connected subnetwork containing 244 targets (83.28% of total) as the CLD disease background network.

This CLD network was initially clustered into 16 modules by ReactomeFIViz (Wu and Haw, 2017). After removing the two modules fully composed of background bridging nodes, we kept the remaining 14 modules for further analysis.

### Common and Unique Functional Modules for TCM Formulae

Since active ingredient list is often an arguable issue for herbs, consensus has not been reached particularly for those commonly found among herbs, such as kaempferol, vitamins, or quercetins. Despite the molecular activity *in vitro*, the low bioavailability was often reported (Barve et al., 2009). Under the complexity of multi-ingredients context of herbs, their potential ADME effect to known active ingredients deserves further investigation. Taking kaempferol as an example, we tested the effects of kaempferol on the absorption of astragalosides in Huangqi herb *via* verted rat sac method under 4 different dosages. In the preliminary test, it was found that there were significant differences in the absorption of astragalosides in different rats and different gut segments. Then, Latin square was adopted to exclude the influence of different rats and intestinal segments. As the detailed results shown in **Supplementary Table 3**, under the concentration range of 1-10µM, kaempferol was observed with no significant effect on the absorption of 10 components in astragalosides in verted rat sac model.

As such, the active ingredient list was perturbed from full to shortlist by removing kaempferol, vitamin C, and vitamin E one by one. The module enrichment results were summarized in **Supplementary Table 1** (Sheet name: results of enrichment). Despite the perturbation, all formulae share 3 consensus modules and one conditional module, and each formula own two unique modules. Ten modules covered 236 targets, and their functional annotation are shown in **Table 1** by GeneCards (Safran et al., 2010).

**Table 1:** List of functional modules for CLD disease network derived from three TCM formulae.

Module ID	Module Function
1	Immune Response (lectin induced complement pathway)
2	ECM-receptor interaction; Leukocyte trans-endothelial migration Fluid shear stress and atherosclerosis; Cell adhesion
3	ATP Binding (LKB1 signaling events)
4	CDK-mediated phosphorylation and removal of Cdc6
5	ATP synthesis; Doxorubicin Pathway (Cardiomyocyte Cell), Respiratory electron transport
6	Neurotransmitter Release Cycle
7	Drug metabolism - cytochrome P450 Oxidative Stress
8	DNA Damage Oxidative Stress
9	NFAT and Cardiac Hypertrophy
10	Jak-STAT signaling; Cytokine-cytokine receptor interaction IL-17 signaling; Inflammatory mediator regulation of TRP channels

**Figure 2:**
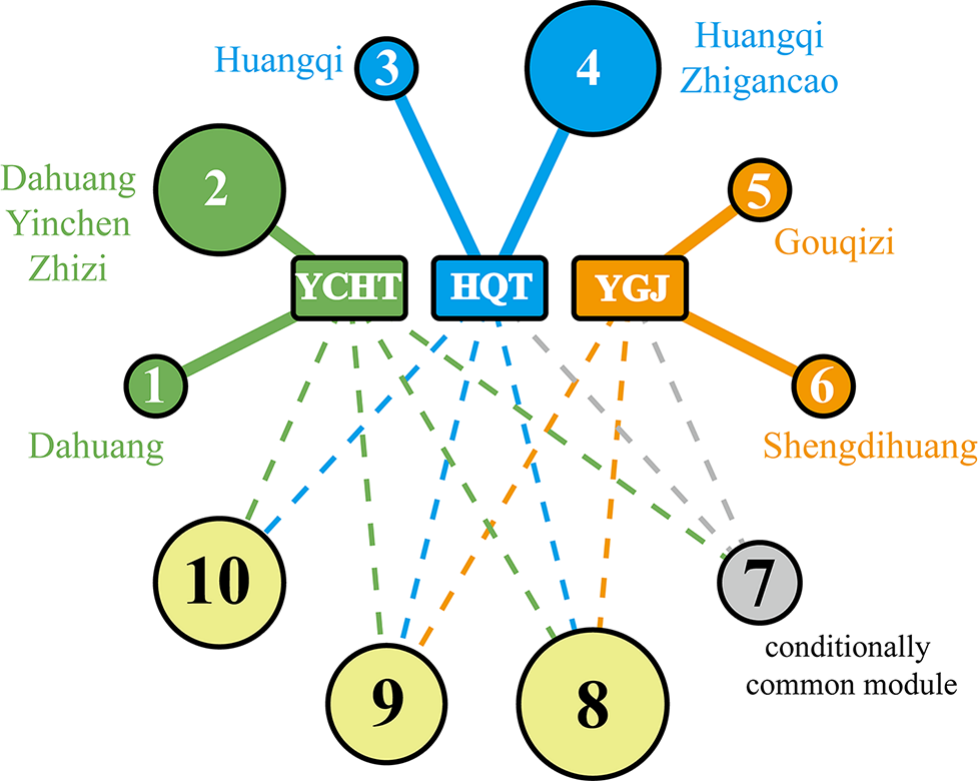
Common and unique functional modules between the three formulae. Each cycle represents a functional module, and the size of each cycle is correlated to the number of genes in each module. Modules 8, 9, and 10 are illustrated as common module, while module 7 is meant as conditional common module. Herbs are labelled as well indicating that their ingredients can target unique module.

**Figure 3:**
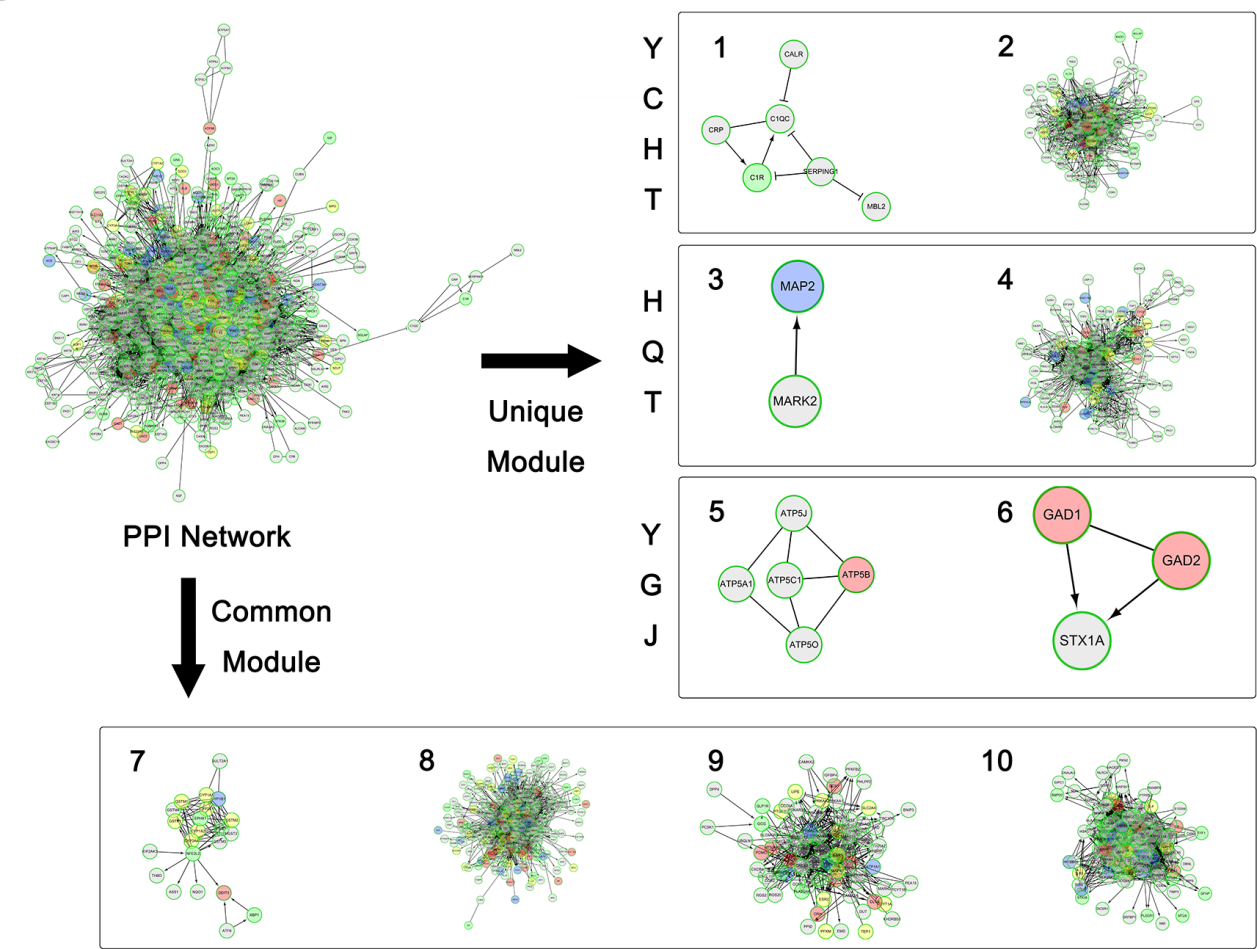
The overall network modularization. Green nodes indicate unique targets of YCHT; blue nodes indicate unique targets of HQT; pink nodes indicate unique targets of YGJ; and yellow nodes indicate overlapping targets from 2 or 3 formulae.

Through the computational framework, the MOA similarity and differences are illustrated in **Figure 2** as shown below in the background of disease modules. The four common modules cover 167 targets, mainly involving oxidative stress, drug metabolism, DNA damage, NFAT and Cardiac Hypertrophy, Jak-STAT signaling and inflammation, etc. Further details of modules are shown in **Figure 3**. These common processes of oxidative stress, inflammation, and corresponding signaling pathways are highly involved in the pathological basis of CLD. For instance, oxidative stress, caused by imbalance between production and consumption by antioxidants, is a prominent feature in the pathophysiology of CLD (Vuppalanchi et al., 2011). Once oxidative stress is initiated, continuous cycle of cellular damage and release of proinflammatory cytokines will move on leading to hepatic inflammation, fibrosis, and cirrhosis (Comporti et al., 2009).

In view of literature report, consistent mechanistic evidences have been found between our computing analysis and independent validation. Modules 8 and 7 are involved with anti-oxidative stress. All three formulae can target these two common modules, indicating their common anti-oxidative function. Furthermore, module 10 is mainly involved with inflammation, and all three formulae were predicted to interact with a wide variety of inflammation factors. For instance, YCHT can target IL1B, IL4, and IL6; HQT can target IL6 and IL10; and YGJ can target more factors of IL1B, IL2, IL4, IL5, IL6, IL10, and IL13. At molecular level, our predictive results gained support from previous validation in that TGF-beta was targeted by HQT, and collagen genes (COL1A1, COL4A2, and COL7A1) were targeted by YGJ.

Meanwhile, each formula can significantly target two unique modules as illustrated in **Figure 2**. YCHT can significantly target modules 1 and 2. Module 1 is specifically involved with immune response of lectin induced complement pathway, where Dahuang herb can uniquely interact with it. Module 2 is related to leukocyte trans-endothelial migration and cell membrane interaction or adhesion, where all herbs in YCHT can target this module. HQT compounds can interact with CDK-mediated phosphorylation and removal of Cdc6, together with ATP binding in LKB1 signaling events (modules 3 and 4). Most interestingly, YGJ ingredients can directly and uniquely target the ATP synthesis (Kamanna et al., 2013) and neurotransmitter release cycle (modules 5 and 6), implying the potential modulation of ATP energy and neuron alertness.

### Different Regulation Modes in the Same Functional Module

It is noted that, despite the unique modules for different formulae, the majority of targets are still involving the same functional modules. We investigated the regulation modes of herbal ingredients in each formula on the same module and found that a substantial number of targets are differently regulated by different formulae. It’s realized that the information of regulation modes on targets is highly precious, where only a few databases such as HIT collected the sparse evidences. Details of regulation modes on three consensus modules can be found in **Table 2**.

**Table 2:** Different regulation modes of different formulae on the same functional module. Information of regulation modes is collected from HIT database (Ye et al., 2011).

8	YCHT	da huang	rottlerin	Activate	SOD3	P08294
		yin chen	salicylic acid	Activate	SOD1	P00441
	HQT	huang qi	acetic acid	Inhibit	SOD1	P00441
		huang qi	lupeol	Inhibit	SOD1	P00441
	YGJ	chuan lian zi	oleic acid	Activate	SOD1	P00441
		dang gui	z-ligustilide	Activate	SOD1	P00441
						
9	YCHT	da huang	emodin	Inhibit	ESR1	P03372
	HQT	huang qi	lupeol	Activate	ESR1	P03372
	YGJ	bei sha shen	apigenin	Inhibit	ESR1	P03372
		bei sha shen	psoralen	Activate	ESR1	P03372
						
10	YCHT	da huang	emodin	Activate	IL1B	P01584
		da huang	emodin	Inhibit	IL1B	P01584
		da huang	rottlerin	Inhibit	IL6	P05231
		yin chen	salicylic acid	Inhibit	IL4	P05112
	HQT	huang qi	acetic acid	Inhibit	IL10	P22301
		huang qi	saikosaponin-d	Inhibit	IL6	P05231
	YGJ	bei sha shen	apigenin	Inhibit	IL4	P05112
		bei sha shen	apigenin	Inhibit	IL13	P35225
		bei sha shen	apigenin	Inhibit	IL2	P60568
		bei sha shen	lauric acid	Activate	IL6	P05231
		bei sha shen	methyl palmitate	Activate	IL10	P22301
		bei sha shen	methyl palmitate	Activate	IL6	P05231
		bei sha shen	methyl palmitate	Inhibit	IL10	P22301
		bei sha shen	sesamol	Activate	IL10	P22301
		bei sha shen	styrene	Inhibit	IL4	P05112
		bei sha shen	styrene	Activate	IL4	P05112
		bei sha shen	styrene	Activate	IL5	P05113
		bei sha shen	styrene	Activate	IL13	P35225
		chuan lian zi	ginkgolide b	Inhibit	IL1B	P01584
		dang gui	palmitic acid	Activate	IL10	P22301
		sheng di huang	gamma-aminobutyric acid	Inhibit	IL6	P05231

For the module of DNA Damage and Oxidative Stress (Module 8), we can see that YCHT and YGJ ingredients can activate the antioxidant-related genes SOD families, while HQT compounds inhibit SOD1 genes. This actually agrees well with previous literature results for HQT (Wang et al., 2008). On Module 9 of NFAT and Cardiac Hypertrophy, YCHT ingredient can inhibit ESR1 target and HQT ingredient has inverse action, while YGJ shows mixed function. The 10th Module contains a list of inflammation factors. Generally, all formulae inhibit a wide range of pro-inflammation factors, such as IL1, IL2, IL4, IL5, etc.

Previous studies have shown that YCHT can regulate Kupffer cells; inhibit the release of proinflammatory cytokines, such as TNF-α, IL-1β, and IL-6; and promote the release of anti-inflammatory and anti-fibrogenic factors, IFN-γ and IL-10 (Zhang et al., 2013). Palmitic acid contained in herb Zhizi can inhibit the cytokines involved in various inflammatory processes as an antagonist of CASP1 (Xu et al., 2016). Further, the active ingredients in YCHT, such as salicylic acid, rottlerin, and garlic acid can also reduce the expression of NOS2, TNF, and RELA, and then inhibit the inflammation (Hee Sun et al., 2008). Among the cytokines, IL10 is well known for its balanced function of inflammation-inhibition. On this particular gene of IL10, HQT shows negative effects, while YGT ingredients show positive effects. It is noted that only individual ingredients were reported with activating/inhibiting effect on molecular targets; the final *in vivo* effects may depend on the relative content and bioavailability of multiple ingredients in formula.

## Discussion

Highly personalized prescriptions of different formulae are frequently applied to treat one same disease in different states. Comparing the MOA similarities and differences between formulae for same disease will provide us a good opportunity to study the pathogenesis of disease subtypes. However, as shown in this study, the commonly simple adoption of KEGG or GO annotation often gives more similarities than differences because the pathway maps or GO terms have been strictly predefined while they are actually densely cross-talked. We thus set up a computational framework of comparative network pharmacology for this purpose and illustrated an example to reveal MOA details of 3 herbal recipes for CLD disease.

In applying this framework to other formulae, there are some suggestions to get better understanding of the method. First, instead of the predicted targets, we strongly support to collect those clearly validated ingredients and targets with regulation modes. One reason is that the prediction error may expand and mess up the modularization part during the subsequent analysis. Another reason is, regulation modes are normally deficient in the results of computational prediction. That is why we have to abandon several elegant TCM databases, such as SymMap (Wu et al., 2018), TCMSP (Ru et al., 2014), or YaTCM (Li et al., 2018). Secondly, other databases or household PPI list can also be adopted to generate PPI network if the information in Reactome is not enough. Lastly, the statistics of module enrichment is elaborated here by comparing with a randomly picked module with equal size of targeting groups in disease network background.

The comparative analysis finds the MOA difference correlates well with the Zheng symptoms between the formulae. As we know, YCHT is used to treat CLD patients with jaundice or inflammation. The unique function module of YCHT is just related to lectin-induced complement pathway and leukocyte trans-endothelial migration, which might mobilize extra anti-inflammation factors to deal with “damp-heat” symptoms. Another interesting finding here is that YGJ can directly target ATP synthesis and neurotransmitter release, which may promote ATP supply and increase the alertness of neuro systems. In our previous study, YGJ was found with ability to modulate the abnormal energy metabolism (Yan et al., 2017). This unique MOA of YGJ agrees well with its clinical application to treat “Yin deficiency” patients with extreme fatigue and weakness.

Yet, most of the time, disease subtypes of TCM Zheng are classified by highly summarized concepts of tongue color, pulse feeling, and personal overall phenotypes. While the Zheng research is still at beginning stage, our comparison framework from formula's perspective may provide alternative views to study their material basis. In addition to above, more consistent evidence may be proposed in future with both the progress in herbal target information and the TCM Zheng study.

Lastly, interaction between compounds and protein targets plays an important role in modulating the biological process and activity. We collected only those compounds with target information and activity evidence for network analysis. Yet, what ingredients are actually pharmacologically active deserves further investigation for these formulae. Correspondingly, perturbation tests are necessary to reach consensus conclusion. In fact, the list of active compounds may be affected by multiple factors such as the quantities in herbs, herb dosage, conjugated forms, compound interactions, biotransformation, dietary usage, bioavailability, other ADME issues (Dabeek and Marra, 2019).

To summarize, the comparative network pharmacology can detect a list of functional modules inferring the potential mechanisms of different formulae and provide functional insights for the subsequent exploration for not only formulae, but also disease subtyping. It's aware that all the information in the framework is collected from public database. Thus, the analysis results may be influenced by the abundancy and accuracy of related information. In the future, with more herbal experimental evidences accumulated, MOA comparison between different formulae is expected to be refreshed quantitatively and highlighted.

## Data Availability Statement

Publicly available datasets were analyzed in this study. This data can be found here: www.ncbi.nlm.nih.gov/pmc/articles/PMC3013727/.

## Ethics Statement

The experimental procedures involving the use of animals were approved by the Committee on the Use of Live Animals for Teaching and Research of the Shanghai University of Traditional Chinese Medicine and all experiments were performed in accordance with the guidelines of this committee.

## Author Contributions

ZKC conducted the project and wrote the manuscript. YL and YW finished the experimental verification. XW, KT, and DW collected the data and interpreted the results. WZ modified the manuscript. YM, PL, and ZWC designed and supervised the project. All authors read, critically reviewed, and approved the final manuscript.

## Funding

This work was supported in part by National Key R&D Program of China (grant number 2017YFC1700200 and 2017YFC0908400), the National Natural Science Foundation of China (31671379, 81573873, 81774196).

## Conflict of Interest

The authors declare that the research was conducted in the absence of any commercial or financial relationships that could be construed as a potential conflict of interest.

## Supplementary Material

Supplementary Table 1 The detailed results of targets and ingredients.

Supplementary Table 2 The detailed results of Enrichment in KEGG and GO.

Supplementary Table 3 The detailed results of the everted intestinal sacs experiment.
